# Predictive Value of MRI in Hypoxic-Ischemic Encephalopathy Treated with Therapeutic Hypothermia

**DOI:** 10.3390/children10030446

**Published:** 2023-02-25

**Authors:** Alessia Guarnera, Giulia Lucignani, Chiara Parrillo, Maria Camilla Rossi-Espagnet, Chiara Carducci, Giulia Moltoni, Immacolata Savarese, Francesca Campi, Andrea Dotta, Francesco Milo, Simona Cappelletti, Teresa Capitello Grimaldi, Carlo Gandolfo, Antonio Napolitano, Daniela Longo

**Affiliations:** 1 Neuroradiology Unit, Imaging Department, Bambino Gesù Children’s Hospital IRCCS, Piazza Sant’Onofrio, 4, 00165 Rome, Italy; 2 Neuroradiology Unit, NESMOS Department Sant’Andrea Hospital, La Sapienza University, Via di Grottarossa, 1035-1039, 00189 Rome, Italy; 3 Medical Physics Unit, Risk Management Enterprise, Bambino Gesù Children’s Hospital IRCCS, Piazza Sant’Onofrio, 4, 00165 Rome, Italy; 4 Neonatal Intensive Care Unit, Department of Medical and Surgical Neonatology, Bambino Gesù Children’s Hospital, IRCCS, Piazza Sant’Onofrio, 4, 00165 Rome, Italy; 5 Unit of Clinical Psychology, Department of Neuroscience, Bambino Gesù Children’s Hospital, IRCCS, Piazza Sant’Onofrio, 4, 00165 Rome, Italy

**Keywords:** hypoxic-ischemic encephalopathy, HIE, perinatal asphyxia, MRI, ADC, van Rooij, prognostic parameter, early biomarker, ROC curve

## Abstract

Background: Hypoxic-ischemic encephalopathy (HIE) is a severe pathology, and no unique predictive biomarker has been identified. Our aims are to identify associations of perinatal and outcome parameters with morphological anomalies and ADC values from MRI. The secondary aims are to define a predictive ADC threshold value and detect ADC value fluctuations between MRIs acquired within 7 days (MR0) and at 1 year (MR1) of birth in relation to perinatal and outcome parameters. Methods: Fifty-one term children affected by moderate HIE treated with hypothermia and undergoing MRI0 and MRI1 were recruited. Brain MRIs were evaluated through the van Rooij score, while ADC maps were co-registered on a standardized cerebral surface, on which 29 ROIs were drawn. Statistical analysis was performed in Matlab, with the statistical significance value at 0.05. Results: ADC0 < ADC1 in the left and right thalami, left and right frontal white matter, right visual cortex, and the left dentate nucleus of children showing abnormal perinatal and neurodevelopmental parameters. At ROC analysis, the best prognostic ADC cut-off value was 1.535 mm^2^/s × 10^−6^ (sensitivity 80%, specificity 86%) in the right frontal white matter. ADC1 > ADC0 in the right visual cortex and left dentate nucleus, positively correlated with multiple abnormal perinatal and neurodevelopmental parameters. The van Rooij score was significantly higher in children presenting with sleep disorders. Conclusions: ADC values could be used as prognostic biomarkers to predict children’s neurodevelopmental outcomes. Further studies are needed to address these crucial topics and validate our results. Early and multidisciplinary perinatal evaluation and the subsequent re-assessment of children are pivotal to identify physical and neuropsychological disorders to guarantee early and tailored therapy.

## 1. Introduction

Hypoxic-ischemic encephalopathy (HIE) is a severe pathology affecting 0.5–1/1000 live births in Western countries, presenting a mortality rate varying between 10 and 60% and neurodevelopmental sequelae affecting nearly 23% of children [1,2,3]. Perinatal asphyxia is the most frequent cause of HIE [1,4], and the diagnostic pathway to identify indications for therapeutic hypothermia encompasses newborn sequential assessment using three criteria related to clinical and laboratory parameters, Sarnat score, and electroencephalography recording [5,6]. If therapeutic hypothermia is indicated, and no exclusion parameters are present, patients undergo therapeutic hypothermia within 6 h, which consists of cooling the neonate at 33.5 for 72 h, followed by progressive heating at 0.25–0.5 °C/h over the next 12 h [1,4,6].

Literature has shown that therapeutic hypothermia can improve patients’ prognoses and has become routine practice in the field of therapy for HIE [1,3,7]. Various MRI biomarkers with various cut-off levels have been suggested to predict neurodevelopmental outcomes, although no univocal results have been obtained [8,9,10,11].

Identifying early and non-invasive biomarkers to predict children’s prognoses and neurodevelopmental outcomes is particularly complicated in children with moderate disease, and it is crucial to provide fact-based awareness to worried parents and to plan an early, multidisciplinary, and tailored treatment for patients [12,13,14,15].

The primary objectives are to identify associations between both the neurodevelopmental outcome of hypoxic-ischemic encephalopathy at 1 year and the perinatal clinical-laboratory parameters with (a) brain anomalies in MRI, scored by van Rooij et al. [16], and (b) ADC values in brain areas sensitive to hypoxic-ischemic injury.

The secondary objectives are (c) to identify an ADC threshold value to differentiate patients in relation to their prognosis; (d) to detect fluctuations in brain ADC values between MRIs acquired within 7 days (MRI0) and at 1 year (MRI1) of life; and (e) to correlate fluctuations in ADC values with perinatal and neurodevelopmental parameters.

## 2. Materials and Methods

Our retrospective observational study was conducted in accordance with the 1964 Helsinki Declaration and its later amendments or comparable ethical standards. The study was approved by the Institutional Review Board and the Research Ethics Committee of the Bambino Gesù Hospital of Rome (approval code: 1917_OPBG_2019; date: 24 July 2019). Written informed consent was obtained from the parents of all individual participants included in the study before MRI scans and before therapeutic procedures.

### 2.1. Participants

Patients were retrospectively and consecutively recruited from the archive of our institution by a neonatologist with 30 years of experience in the time frame 1 January 2013 to 30 June 2020. Inclusion criteria encompassed: gestational age between 36 and 40 weeks, diagnosis of HIE made at birth, therapeutic hypothermia performed within 6 h of birth, and high-quality MRI performed within 7 days and at 1 year of birth, including at least T2WI and DWI/ADC sequences. The parameters used to define high-quality MRIs were as follows: absence of motion degradation, absence of significant artifacts that could have impaired the exam evaluation, and sequences covering the entire brain. Exclusion criteria were: significant comorbidities, such as intracranial or systemic tumors, infections, or severe malformations; maternal risk factors, such as drug or alcohol abuse; and incomplete data, including perinatal clinical data, laboratory data, and neurodevelopmental data at 1 year.

The following patients’ data were collected and reported in an anonymized database: demographic characteristics; gestational age; eutocic or dystocic delivery (both the causes of maternal and child dystocia, including the need to use forceps or suction cups and invasive gynecological maneuvers); perinatal therapies; perinatal complications (need for cardiac massage and/or mechanical ventilation, development of respiratory distress syndrome/RDS); perinatal laboratory values (umbilical cord pH, lactates in the blood); ten-minute Apgar score; children’s neurodevelopment at 1 year, scored using the Bayley III coded scale [17]; and qualitative indices of patients’ suffering, such as sleep disorders and physiokinesitherapy, at 1 year.

**Figure 1:**
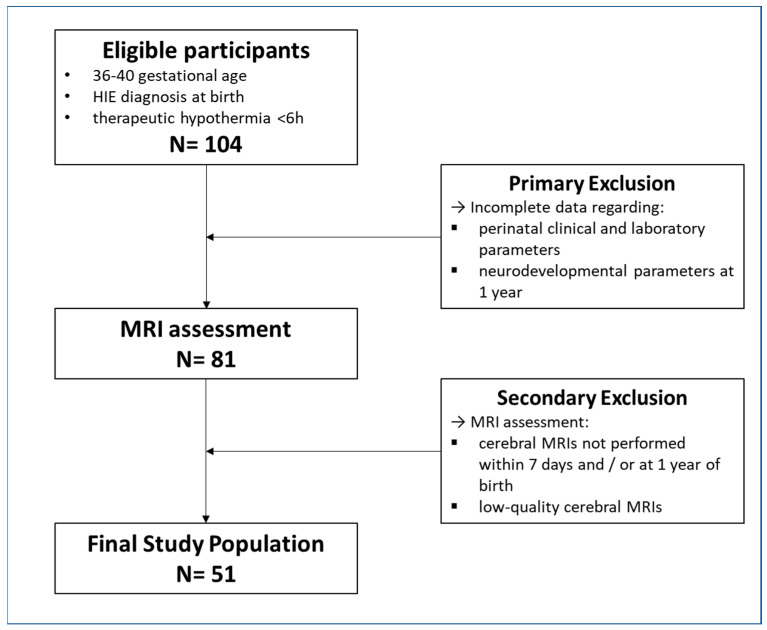
Flow chart showing the final study population selection.

The final cohort of patients consisted of 51 subjects presenting with moderate HIE (Figure 1).

### 2.2. MRI Acquisition

Newborns were either placed on their backs in carrying mats (Medical VAC), which can ensure the maintenance of the position and avoid motion artifacts or were sedated. Infants requiring mechanical ventilation and/or anticonvulsant therapy received an increase in sedative and/or anticonvulsant dosage before the scan. Ear protectors were placed in patients’ ears before scanning. The temperature was kept stable, and vital signs, such as heart rate and oxygen saturation, were monitored by a pediatric anesthesiologist with 30 years of experience.

Brain MRIs were performed within 7 days and at 1 year from birth, following therapeutic hypothermia, on the same 3T scanner (Magnetom Skyra, Siemens, Erlangen, Germany) with a 32-channel brain coil (L-W-H: 440 mm 330 mm 370 mm) and using the following protocol: axial TSE T2 (TR 6380 ms, TE 109 ms, ST 3 mm); coronal TSE T2 (TR 6380 ms, TE 109 ms, FA 150, ST 3 mm); axial DWI (TR 6400 ms, FA 98 ms, FA 75 m); and 3D T1 MPRAGE (TR 1570 ms, TE 2.67 ms, TI 900 ms, FA 9 laying, ST 0.8 mm).

### 2.3. Radiological Evaluation

#### 2.3.1. Van Rooij Score

Brain MRIs were evaluated and scored with the van Rooij score [16] by two pediatric neuroradiologists with 15 and 10 years of experience. Any discrepancies were solved by a pediatric neuroradiologist with 30 years of experience. Patients were divided into two groups in relation to the presence of signal abnormalities.

#### 2.3.2. Measurement of ADC Values

ADC maps were generated from DWI sequences in all newborns and then co-registered on a standardized cerebral surface, which is widely used in pediatric studies [14]. On the standardized cerebral surface, a pediatric neuroradiologist with 10 years of experience manually drew 29 regions of interest (ROI) in brain areas sensitive to hypoxic-ischemic injury (Supplementary Materials Table S1 and Figure S1). The correct positioning of the ROIs was visually evaluated and, if needed, corrected by the same neuroradiologist before quantitative data extraction from the maps of ADC. ROIs vary in size and morphology, depending on brain structures and to avoid CSF inclusion. However, directly drawing the ROIs on the standardized cerebral surface on which all the ADC maps were co-registered eliminated inter-individual variability and guaranteed the homogenization of ROI positioning, morphology, and area among the patients.

### 2.4. Statistical Analysis

Statistical analysis was performed in Matlab, with the statistical significance value at 0.05.

To identify correlations of both patients’ perinatal parameters and neurodevelopmental outcomes with imaging findings from the MRI acquired within 7 days scored by the van Rooij score, a logistic regression analysis was performed; to identify associations of both patients’ perinatal parameters and neurodevelopmental outcomes with ADC values measured in the ROIs, we performed a linear regression analysis.

Receiver operating characteristic (ROC) analyses were realized for each ROI in relation to the perinatal clinical and laboratory parameters and in relation to the neurodevelopmental parameters at 1 year. The ADC threshold value to differentiate patients in relation to their prognosis was calculated by using the maximum Youden J index.

For longitudinal analysis, ADC0 (ADC of MRI0) and ADC1 (ADC of MRI1) were firstly regrouped by each perinatal and neurodevelopmental parameter. To detect fluctuations in brain ADC values between MRI0 and MRI1, we compared ADC0 and ADC1 values for each ROI using a paired *t*-test. To identify possible ADC differences in MRIs acquired at 1 year of life among subjects in relation to perinatal and neurodevelopmental parameters, a linear regression analysis was performed.

## 3. Results

The final cohort of patients consisted of 51 subjects presenting moderate HIE (Figure 1). Patients’ demographic data and perinatal and outcome parameters were collected and reported in an anonymized database (Table 1).

### 3.1. Correlations between Perinatal and Outcome Parameters with Van Rooij Score at MRI0

In patients presenting with signal abnormalities at MRI0, the median van Rooij score was 3.1 ± 2.7 (SD/standard deviation).

No significant correlations were found between the van Rooij score and the perinatal parameters.

A significant correlation was identified between the van Rooij score and the evidence of sleep disorders (*p* < 0.014).

### 3.2. Correlations between Perinatal and Outcome Parameters with ADC Values at MRI0 and ROC Analysis

ADC0 < ADC1 in the left and right thalami, in the right visual cortex, in the left dentate nucleus, and in the left and right frontal white matter for multiple outcomes and perinatal parameters (Table 2).

ADC0 < ADC1 in the left thalamus in patients presenting with sleep disorders (*p* < 0.026), showing motor abnormalities at Bayley III (*p* < 0.015), and needing physiokinesitherapy (*p* < 0.044); in the right thalamus in patients presenting with sleep disorders (*p* < 0.041) and showing motor abnormalities at Bayley III (*p* < 0.021); in the left frontal white matter in patients presenting with sleep disorders (*p* < 0.022) and showing motor abnormalities at Bayley III (*p* < 0.014); and in the right frontal white matter (*p* < 0.046) and in the left dentate nucleus in patients presenting with motor abnormalities at Bayley III (*p* < 0.028) (Table 2).

**Figure 2:**
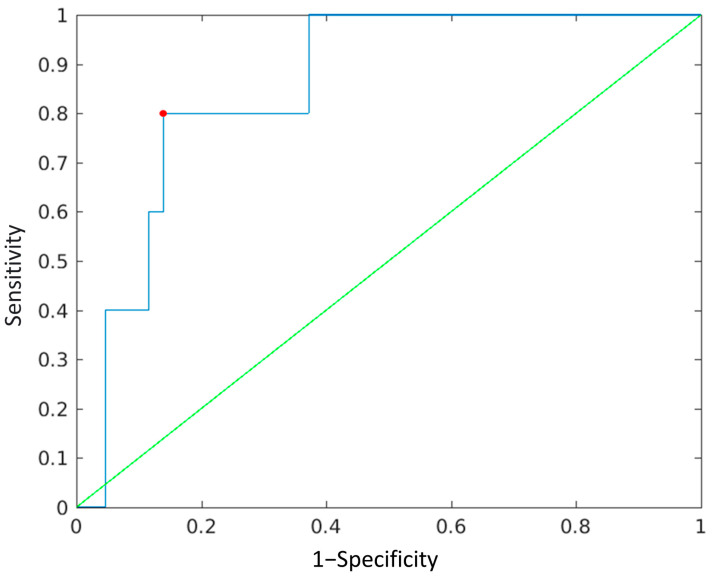
ROC curve for the ADC cut-off value of 1.535 mm^2^/s × 10^−6^ in the ROI drawn in the right frontal white matter for the outcome parameter sleep disorders. The *x*-axis refers to the sensitivity, the y axis refers to 1-specificity. The blue line represents the AUC, while the green line represents the diagonal. For the cut-off value of 1.535 mm^2^/s × 10^−6^, the sensitivity was 80%, and the specificity was 86% (red dot), while the AUC was 0.856.

In ROC analysis and for the cut-off of 1.535 mm^2^/s × 10^−6^ in the ROI drawn in the right frontal white matter, the outcome parameter with the highest area under the curve (AUC) (0.856) was the presence of sleep disorders at 1 year, presenting a sensitivity of 80% and a specificity of 86% (Figure 2).

### 3.3. Fluctuations in ADC Values between MRI0 and MRI1 and Their Correlation with Perinatal and Outcome Parameters

ADC1 > ADC0 across the ROIs, and it was observed that there was a significant value increase in ADC1 > ADC0 in the right visual cortex and the left dentate nucleus for both multiple outcomes and perinatal parameters (Table 2).

## 4. Discussion

We found some significant correlations between perinatal and outcome parameters and imaging. In particular, the van Rooij score was significantly higher in children presenting with sleep disorders at 1 year of life, and ADC0 was significantly lower in children showing abnormal perinatal parameters and compromised neurodevelopmental outcomes in the left and right thalami, left and right frontal white matter, right visual cortex, and left dentate nucleus. The optimal ADC cut-off value was identified in the right frontal white matter (value: 1.535 mm^2^/s × 10^−6^; sensitivity: 80%; specificity: 86%). ADC1 values were higher compared to ADC0 in the right visual cortex and left dentate nucleus and positively correlated with multiple abnormal perinatal parameters and compromised neurodevelopmental outcomes.

### 4.1. Correlations between Perinatal and Outcome Parameters with Van Rooij Score and with ADC Values at MRI0, and ROC Analysis

Results showed that ADC1 values were lower: in the left and right thalami, in the left and right frontal white matter, in the right visual cortex of patients presenting with abnormal perinatal parameters, RDS at birth, and needing cardiac massage and/mechanical ventilation; and in the left and right thalami, in the left and right frontal white matter, and in the left dentate nucleus of patients presenting with sleep disorders and motor abnormalities and needing physiokinesitherapy at 1 year of birth.

**Figure 3:**
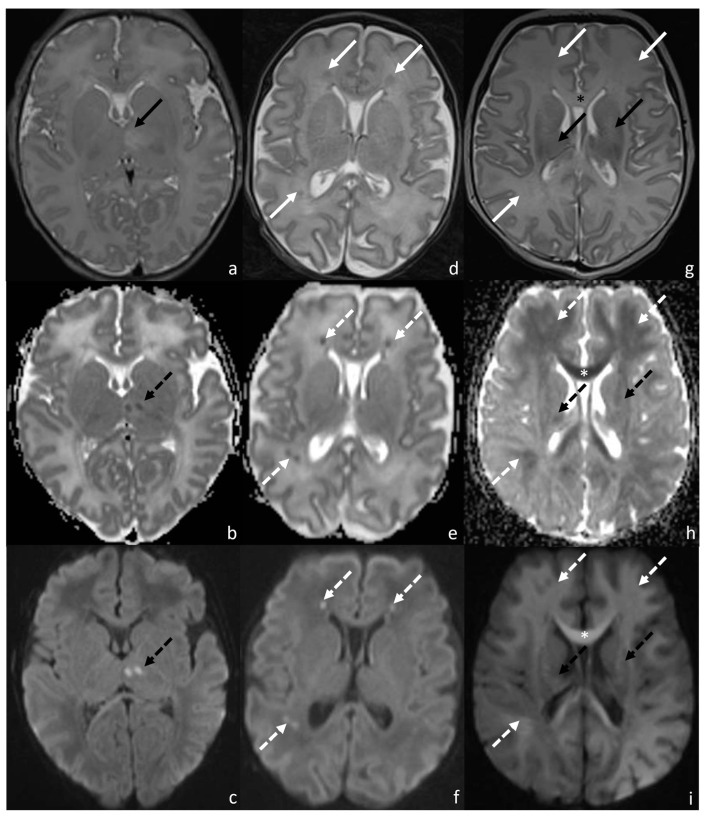
MRI0 of patients affected by HIE injury and presenting: pattern 1 (**a**–**c**), characterized by the prevalent involvement of basal ganglia, thalami, and perirolandic areas; pattern 2 (**d**–**f**), characterized by the prevalent involvement of watershed areas (**c**,**d**); pattern 3, characterized by mixed patterns (**g**–**i**). In particular, the figure shows: T2WI (**a**), ADC (**b**), and DWI (**c**) sequences of patients presenting with pattern 1 and showing areas of diffusion restriction in the left thalamus (dotted black arrow in (**b**,**c**)), paired with focal left thalamic hyperintensity in T2 (black arrow in (**a**)); T2WI (**d**), ADC (**e**), and DWI (**f**) sequences of patients presenting with pattern 2 and showing areas of diffusion restriction in the left and right frontal and right parietal white matter (dotted white arrows in (**e**,**f**)), paired with inhomogenous T2 hypointensity in the same areas (white arrows in (**d**)); T2WI (**g**), ADC (**h**), and DWI (**i**) sequences of patients presenting with mixed patterns showing both areas of diffusion restriction in the capsulo-lenticular regions bilaterally (dotted black arrows in (**h**,**i**)), which match with the focal areas of T2 hyperintensity in the same areas (black arrows in (**g**)); and diffuse areas of diffusion restriction in the corpus callosum (white * in (**h**,**i**)) and in the left and right frontal and right parietal white matter (dotted white arrows in (**h**,**i**)), with aT2 hyperintensity match in the corpus callosum (black * in (**g**)) and no clear evidence of T2 signal alterations in the remaining areas of (**g**).

Term infants affected by HIE usually present with two peculiar patterns on the MRI: the main involvement of basal ganglia, thalami, and perirolandic areas, leading to severe motor impairments, and the involvement of watershed areas, resulting principally in cognitive impairment. Severe HIE forms result in diffuse gray and white matter involvement, characterized by both cognitive and motor impairments [18,19,20,21] (Figure 3).

Most of the perinatal parameters were altered in neonates presenting with low ADC values in the thalami, and this data can be easily explained, since thalami are particularly sensitive to hypoxic-ischemic injury, appearing to be even more vulnerable than basal ganglia to ischemic insult [22,23]. The increased vulnerability was explained by the spreading of hypoxic-ischemic injury from the cortex to the thalami via the axons in the white matter and only secondly to the basal ganglia [24]. Since the DWI may need a few days to reach the complete extent of the lesions [25], thalami showed lower scores, being among the first to suffer from hypoxic-ischemic injury. This data is corroborated by studies on stroke patients, since primary injuries may disrupt functional connections, resulting in the development of secondary injuries, such as thalamic damage, which impair patients’ recovery [26,27].

The hypothesis of injury spreading through the white matter to the thalami was strengthened by the evidence of lower ADC values in the left and right frontal white matter, which was much more evident in severe patients who needed cardiac massage and presented with RDS [24]. The frontal white matter is the site of the pyramidal tract, which arises from the primary motor cortex in the precentral cortex and through the posterior limb of the internal capsule, reaching the bulbar pyramid, where it decussates and continues its pathway in the spinal cord. Neurons of the primary, secondary, and accessory motor cortices receive multiple inputs from different areas of the brain. In particular, significant mediation and modulation are made by the anterior motor-related thalamic regions, including the ventral anterior thalamic and the ventrolateral thalamic nuclei, and the posterior sensory-related thalamic areas, including the posterior thalamic nuclear group [28,29].

Therefore, there was also high consistency between correlations found between ADC0 values and perinatal and outcome parameters at 1 year. In particular, the motor impairment identified by the Bayley III scale optimally correlated with low ADC values identified in the right and left thalami and in the frontal left and right white matter.

Moreover, low ADC values were identified in the dentate nucleus, whose dorsal motor domain controls and regulates motor functions. In particular, the dentate nucleus receives inputs from the premotor and supplementary cortices and sends outputs to the thalami via the dentate–thalamic tract. From the thalami, inputs will be projected to the premotor and primary motor cortices, to the basal ganglia, to the posterior parietal cortex, and to the substantia nigra. Fine movements are regulated by the myoclonic triangle, which consists of the dentate nucleus, the red nucleus, and the inferior olivary nucleus [30,31,32,33]. Consequently, and in accordance with this evidence, low ADC values were scored by patients needing physiokinesitherapy.

The perfect balance between excitatory and inhibitory inputs to the pyramidal tract is impaired in case of insults to the corticospinal tracts or indirectly to the thalami, such as in neonates affected by hypoxic-ischemic injury and presenting with motor impairment at 1 year of birth and justifies the correlation between imaging findings and abnormal neuromotor outcomes.

Low ADC values were also identified in the visual cortex, and that is not surprising, since HIE represents the leading cause of cerebral visual impairment, according to the study by Pehere et al. [34]. The paper proved that HIE caused more than one-third of cerebral visual impairments in children <2 years, the majority of whom presented with severe disability [34].

A significant correlation was found between the van Rooij score and the ADC0 values with sleep disorders. Interestingly, sleep disorders are among the least-investigated symptoms in pediatric populations and are rarely evaluated as outcomes in pediatric patients affected by HIE [35]. However, recent literature demonstrated that neonates affected by HIE showed a delayed onset of the sleep–wake cycle and developed sleep disorders, compromising children’s and parents’ quality of life [35,36,37].

Sleep–wake cycle generation is a complex mechanism involving multiple interconnected cerebral areas and reflects the integrity, maturity, and organization of the neuronal network. Takenouchi et al. [36] showed the abnormalities of sleep–wake cycling at the EEG, reflecting the involvement of the basal forebrain and the cortical, thalamic, and hypothalamic neurons in patients affected by HIE. In particular, the wake-promoting pathways arise from the midbrain and split into the ventral pathway, innervating the basal forebrain, the cortex, and the hypothalamus, and the dorsal pathway, innervating the thalami [38].

Diffuse supratentorial ischemic insult has been frequently related to the disruption of the sleep–wake cycle and justifies the correlation found. Moreover, an increasing amount of evidence demonstrates that the involvement of the thalami and/or the basal ganglia is associated with the delayed onset of sleep–wake cycles and with consequent sleep disorders [36,39]. These data are corroborated by our results since our patients showed low ADC values both in the frontal white matter and in the thalami. Since these areas are crucial in the sleep–wake cycle, hypoxic-ischemic insults disrupt the neuronal network and the related pathways, causing consequent sleep disorders.

Accordingly, sleep disorder was the outcome parameter that most significantly correlated with increased van Rooij scores in the right frontal white matter [9].

Finally, in ROC analysis and for the cut-off of 1.535 mm^2^/s × 10^−6^ in the ROI drawn in the right frontal white matter, the outcome parameter with the highest area under the curve (AUC) (0.856) was the presence of sleep disorders at 1 year, presenting a sensitivity of 80% and a specificity of 86%.

The results of the ROC curve, together with the results of the ADC analysis and the van Rooij score, are particularly interesting since the presence of sleep disorders seems to be the most consistent and relatable parameter investigated, even if this pathology is rarely assessed in HIE patients.

In particular, we only included patients presenting with high-quality MRIs acquired within 7 days. The identification of lower ADC values in MRIs performed within 7 days indicated restricted diffusion in areas that are extremely sensitive to hypoxic-ischemic insult and corresponded with cytotoxic oedema, leading to irreversible lesions secondary to cell death [40]. The MRIs carried out within 7 days from birth allowed for an optimal evaluation of the morphostructural and functional sequences that are affected by defined processes of “pseudonormalization”, or the normalization of the neuroradiological picture that persists after 7 days [9,41]. Therefore, the predictive value of ADC as an early biomarker may be impaired if the scan is performed after 7 days [42].

### 4.2. Fluctuations in ADC Values between MRI0 and MRI1 and Their Correlation with Perinatal and Outcome Parameters

ADC1 values tend to diffusively grow across the ROIs, and a significant increase in ADC1 values compared to ADC0 values in the right visual cortex and the left dentate nucleus was observed for both multiple outcome and perinatal parameters.

In literature, the difference between MRIs acquired within and after 7 days of birth extensively described an increase in ADC values in relation to the pseudonormalization phenomenon [9,41], but few studies investigated the correlation with late MRI appearance [43].

The identification of an increase in ADC values in MRIs performed at 1 year, compared to ADC values performed within 7 days, is related to a persistent and significantly high ADC value. Its correlation with perinatal and outcome parameters at 1 year excludes normalization and suggests, instead, an ischemic scar in different brain areas following HIE.

Accordingly, the correlation of ADC1 values increased with multiple abnormal perinatal and outcome parameters, suggesting that the severity of the HIE is a key factor in defining brain damage. On the other hand, the reduced number of brain areas identified in MRI1, compared to the higher number of areas identified in MRI0, may be explained by the proven effectiveness of therapeutic hypothermia, which reduces cerebral injury burden and improves patients’ prognoses [1,3,7].

The crucial importance of identifying prognostic biomarkers in HIE and of correctly diagnosing and classifying neonates according to the severity of their pathology fueled a vast amount of literature, some of which efficiently applied AI to provide quantitative findings to stage neonates affected by HIE [44]. Our results confirm the vast majority of literature, which, nevertheless, show inhomogeneous results [4,9,23,43,44,45,46]. We hypothesize that the inconsistency that exists among papers stems from the extensive differences in patients’ cohorts in relation to number, severity score, and demographic features; from the absence of a standardized and universally performed MRI protocol; and from the timing of imaging acquisition. Further studies are needed to address these crucial topics and validate our results.

The first limitation of the present study is the absence of a group of patients matched for HIE severity, age, and sex who did not undergo therapeutic hypothermia. Therapeutic hypothermia has been proven to significantly improve pediatric patients’ prognoses, and therefore, it is not ethically acceptable to randomly assign patients to therapy or placebo in case of hypothermia indications. The second limitation is the imbalance between patients with good prognoses and patients presenting with severe prognoses at 1 year. These data are justified by the positive effects of therapeutic hypothermia and by the strict protocols that our institution applied to guarantee early diagnosis and therapy to children.

## 5. Conclusions

ADC values in MRI0 were significantly lower in children showing abnormal perinatal parameters and compromised neurodevelopmental outcomes in the left and right thalami, left and right frontal white matter, right visual cortex, and left dentate nucleus. In particular, at ROC analysis, an ADC threshold value of 1.535 mm^2^/s × 10^−6^, measured in the right frontal white matter, presented a sensitivity of 80% and a specificity of 86% when predicting children’s prognoses at 1 year of life. ADC1 values tended to globally increase, compared to ADC0 values, with particular evidence in the right visual cortex and left dentate nucleus, and were positively correlated with multiple abnormal perinatal parameters and compromised neurodevelopmental outcomes. The van Rooij score was significantly higher in children presenting with sleep disorders at 1 year of life.

Our study highlights the need for a comprehensive MRI study of HIE and underlines the importance of the DWI/ADC sequence for children’s evaluations. ADC values can be used as prognostic biomarkers to predict children’s neurodevelopmental outcomes and provide parents with the most-required information related to patients’ prognoses, which can help them understand and agree with the medical personnel on the best therapy for their children. Further studies are needed to address these crucial topics and validate our results.

Moreover, the results suggest the key importance of early and multidisciplinary perinatal evaluation and subsequent re-assessment of children to identify physical and neuropsychological disorders to guaranteeearly and tailored therapy.

## Figures and Tables

**Table 1:** Main demographic and clinical characteristics of patients affected by hypoxic-ischemic encephalopathy.

Characteristics	Patients
N° of subjects	51
N° F/M	18/33
Eutocic/Dystocic delivery	23/28
Apgar 10 min < 5/≥5	23/28
Blood lactate < 3.9/≥3.9	31/20
Base Excess < −12/≥−12	35/16
Umbilical cord pH < 7/≥7	18/33
Cardiac Massage Y/N	18/33
Mechanical Ventilation Y/N	18/33
RDS	17/34
Bayley III—motion impairment < 85 at 1 year Y/N	6/45
Sleep Disorders at 1 year Y/N	6/45
Physiokinesitherapy at 1 year Y/N	7/44

**Table 2:** Results.

**Results**		
*Correlations between the van Rooij score at MRI0 and perinatal/outcome parameters*		
**Perinatal Parameters**	**Outcome Parameters**	
-	sleep disorders (*p* < 0.0014)	
*Correlations between perinatal and outcome parameters with ADC values at MRI0 and perinatal/outcome parameters*		
**Brain region**	**Perinatal Parameters**	**Outcome Parameters**
left thalamus	pH <7 (*p* < 0.026) BE < −12 mmol/L (*p* < 0.025) lactate <3.9 (*p* < 0.029) RDS (*p* < 0.041) cardiac massage (*p* < 0.032)	sleep disorders (*p* < 0.026) motor impairment (*p* < 0.015) physiokinesitherapy (*p* < 0.044)
right thalamus	lactate <3.9 (*p* < 0.004)	sleep disorders (*p* < 0.041) motor impairment (*p* < 0.021)
right visual cortex	BE < −12 mmol/L (*p* < 0.011) cardiac massage (*p* < 0.047)	-
left frontal white matter	BE < −12 mmol/L (*p* < 0.041) RDS (*p* < 0.038) cardiac massage (*p* < 0.043)	sleep disorders (*p* < 0.022) motor impairment (*p* < 0.014)
right frontal white matter	RDS (*p* < 0.026) cardiac massage (*p* < 0.039)	motor impairment (*p* < 0.046)
left dentate nucleus	-	motor impairment (*p* < 0.028)
*Fluctuations in ADC values between MRI0 and MRI1, and correlation with perinatal and outcome parameters*		
**Brain region**	**Perinatal Parameters**	**Outcome Parameters**
right visual cortex	Dystocic birth (*p* < 0.001) Apgar10 <5 (*p* < 0.001) pHuc <7 (*p* < 0.001) BE < −12 mmol/L (*p* < 0.001) RDS (*p* < 0.001) mechanical ventilation (*p* < 0.001) cardiac massage (*p* < 0,0)	sleep disorders (*p* < 0.001) motor impairment (*p* < 0.007) physiokinesitherapy (*p* < 0.001)
left dentate nuclei	Dystocic birth (*p* < 0.001) Apgar10 (*p* < 0.001) pHuc < 7 (*p* < 0.001) BE < −12 mmol/L (*p* < 0.001) RDS (*p* < 0.001) mechanical ventilation (*p* < 0.001) cardiac massage (*p* < 0.001)	sleep disorders (*p* < 0.009) motor impairment (*p* < 0.007) physiokinesitherapy (*p* < 0.002)

## Data Availability

Data are available from the corresponding author, D.L., upon reasonable request.

## Supplementary Materials

The following supporting information can be downloaded at: https://www.mdpi.com/article/10.3390/children10030446/s1, Figure S1: Twenty-nine ROIs were drawn on the standardized brain surface, specifically on the left and right pre- and post-central cortices (a), frontal and parietal white matters (b), basal ganglia (putamen), posterior limb of the internal capsules, thalami and Heschl’s gyri (c), optic radiations and visual cortices (d), hippocampi, cerebral peduncles and vermis, pontine tegmentum, and dentate nuclei (f). The correct positioning of the ROIs was visually evaluated and, if needed, corrected by a neuroradiologist with 10 years of experience before quantitative data extraction from the maps of ADC. Table S1: Shows the brain region and side on which the ROIs were drawn.

## Abbreviations

HIE: hypoxic-ischemic encephalopathy; RDS: respiratory distress syndrome; MRI: magnetic resonance imaging; MRI0: MRI acquired within 7 days of birth; MRI1: MRI acquired at 1 year of birth; ADC: apparent diffusion coefficient; ADC0: ADC of MRI acquired within 7 days of birth; ADC1: ADC of MRI acquired at 1 year of birth; DWI: diffusion-weighted image; TE: time of echo; ST: slice thickness; TR: time of repetition; TI: inversion time; FA: flip angle; MPRAGE: magnetization-prepared rapid gradient-echo; pH: potential of hydrogen; ROI: region of interest; ROC: receiver operating curve; AUC: area under characteristic; EEG: electroencephalography; SD: standard deviation.
