# Supplementary material for: Predictive Value of MRI in Hypoxic-Ischemic Encephalopathy Treated with Therapeutic Hypothermia
Source: Children (Basel). 2023 Feb 25;10(3):446. doi: 10.3390/children10030446 (PMC10047577; doi:10.3390/children10030446)
Supplement: Supplementary file 1 [file children-10-00446-s001.zip › children-2158750-supplementary.pdf]

ROI n°	Brain Region	Side
1	Basal Ganglia (Putamen)	L
2	Basal Ganglia (Putamen)	R
3	Thalamus	L
4	Thalamus	R
5	Posterior Limb of Internal Capsule	L
6	Posterior Limb of Internal Capsule	R
7	Optic Radiation	L
8	Optic Radiation	R
9	Visual Cortex	L
10	Visual Cortex	R
11	Pre-Central Cortex	L
12	Pre-Central Cortex	R
13	Post-Central Cortex	L
14	Post-Central Cortex	R
15	Frontal White Matter	L
16	Frontal White Matter	R
17	Parietal White Matter	L
18	Parietal White Matter	R

19	Hippocampus	L
20	Hippocampus	R
21	Heschl Gyrus	L
22	Heschl Gyrus	R
23	Vermis	-
24	Dentate Nucleus	L
25	Dentate Nucleus	R
26	Cerebral Peduncle	L
27	Cerebral Peduncle	R
28	Pontine Tegmen	L
29	Pontine Tegmen	R

Supplementary Materials _Table S1. shows the brain region and side on which the ROIs were drawn.

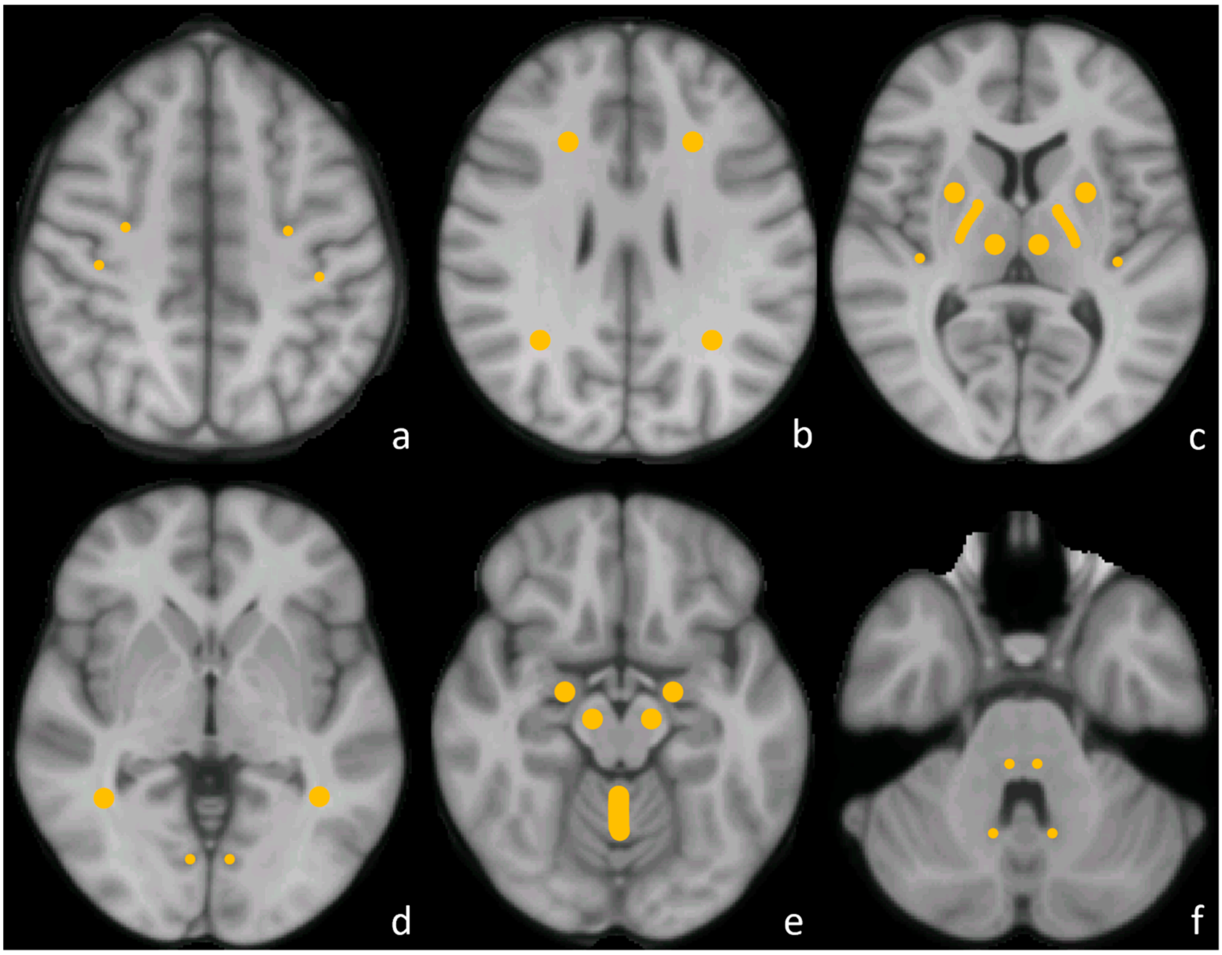

Supplementary Materials _ Figure S1. Twenty-nine ROIs were drawn on the standardized brain surface, specifically on the left and right pre- and post-central cortices (a), frontal and parietal white matters (b), basal ganglia (putamen), posterior limb of the internal capsules, thalami and Heschl's gyri (c), optic radiations and visual cortices (d), hippocampi, cerebral peduncles and vermis, pontine tegmentum, and dentate nuclei (f). The correct positioning of the ROIs was visually evaluated and, if needed, corrected by a neuroradiologist with 10 years of experience before quantitative data extraction from the maps of ADC.
